# Supplementary material for: Development and Validation of a New LC-MS/MS Method for Simultaneous Quantification of Ivacaftor, Tezacaftor and Elexacaftor Plasma Levels in Pediatric Cystic Fibrosis Patients
Source: Pharmaceuticals (Basel). 2025 Jul 10;18(7):1028. doi: 10.3390/ph18071028 (PMC12298270; doi:10.3390/ph18071028)
Supplement: Supplementary file 1 [file pharmaceuticals-18-01028-s001.zip › Supplementary Figures_R1.pdf]

IVACAFTOR

A)

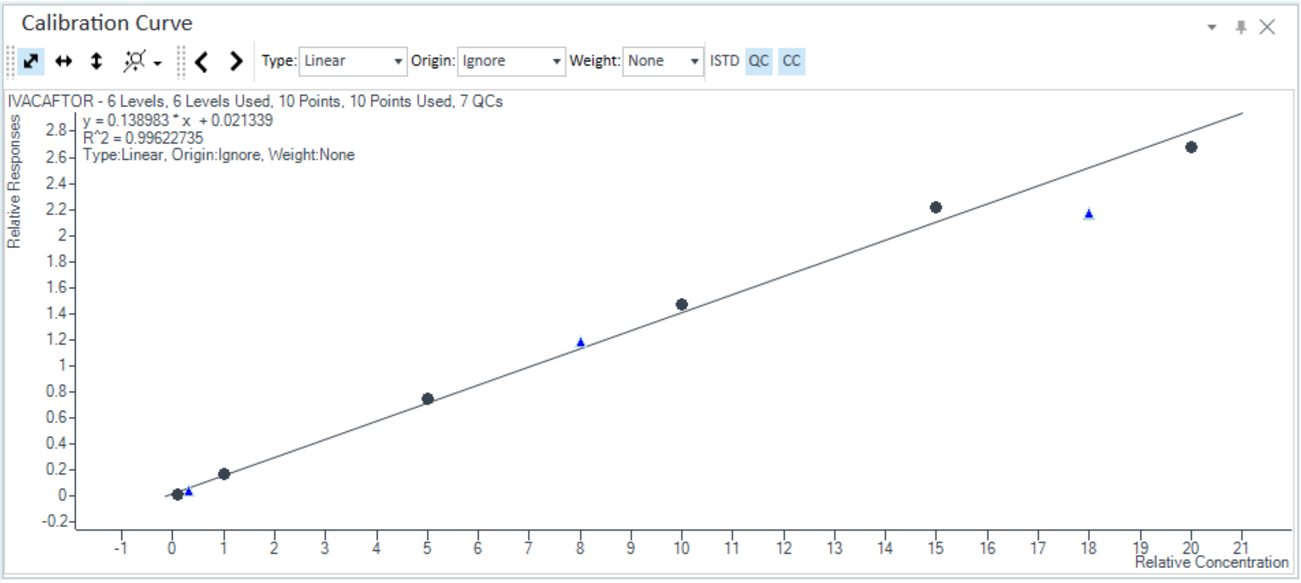

TEZACAFTOR

B)

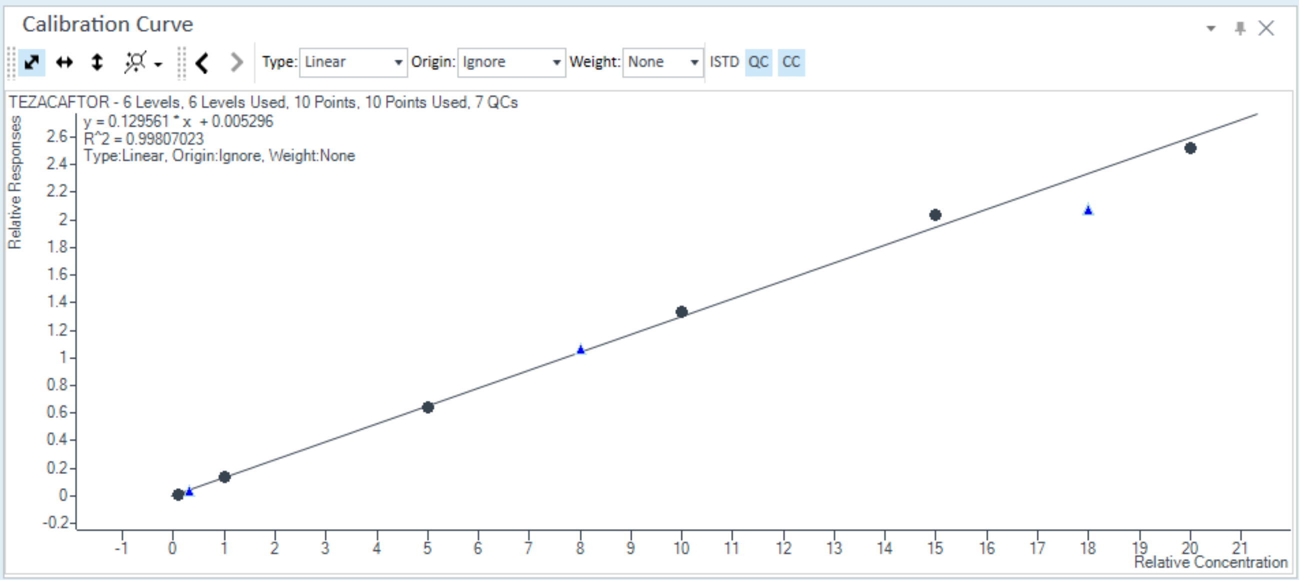

ELEXACAFTOR

C)

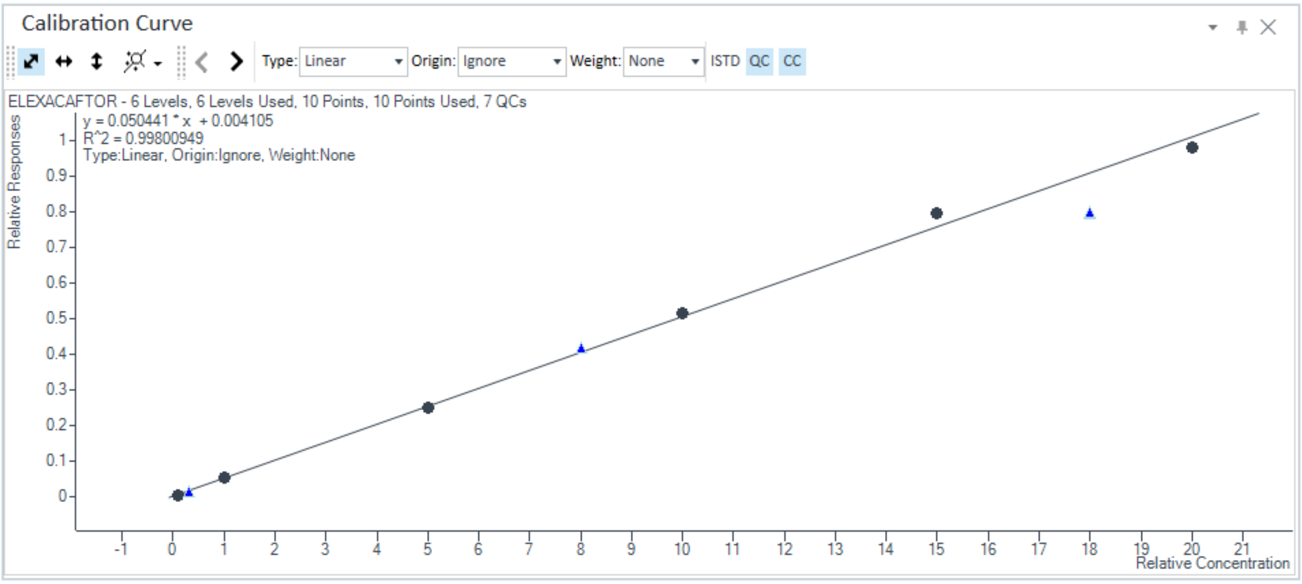

Supplementary Figure 1. Six-point calibration curve for ivacaftor (A), tezacaftor (B) and elexacaftor (C). The calibration curve equation and R2 value are displayed in the inset. Blue triangles indicate L-QC, M-QC, and H-QC samples.

IVACAFTOR

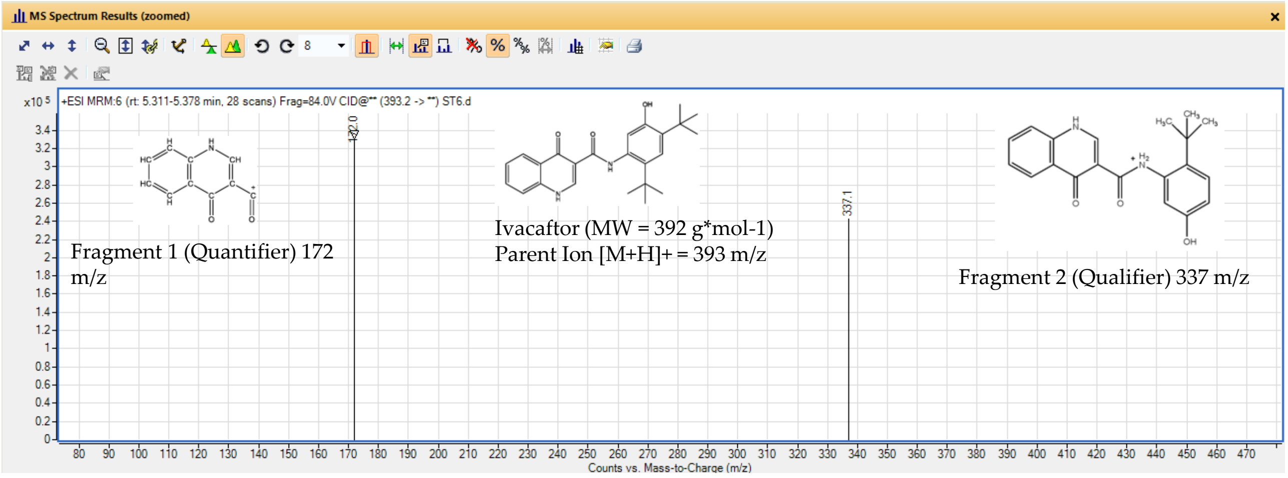

TEZACAFTOR

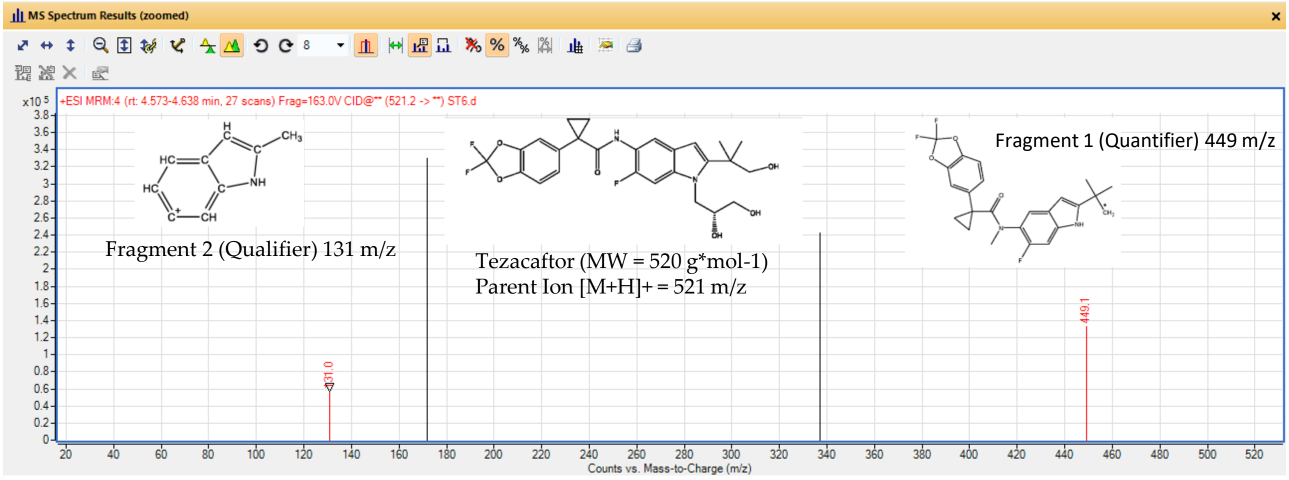

ELEXACAFTOR

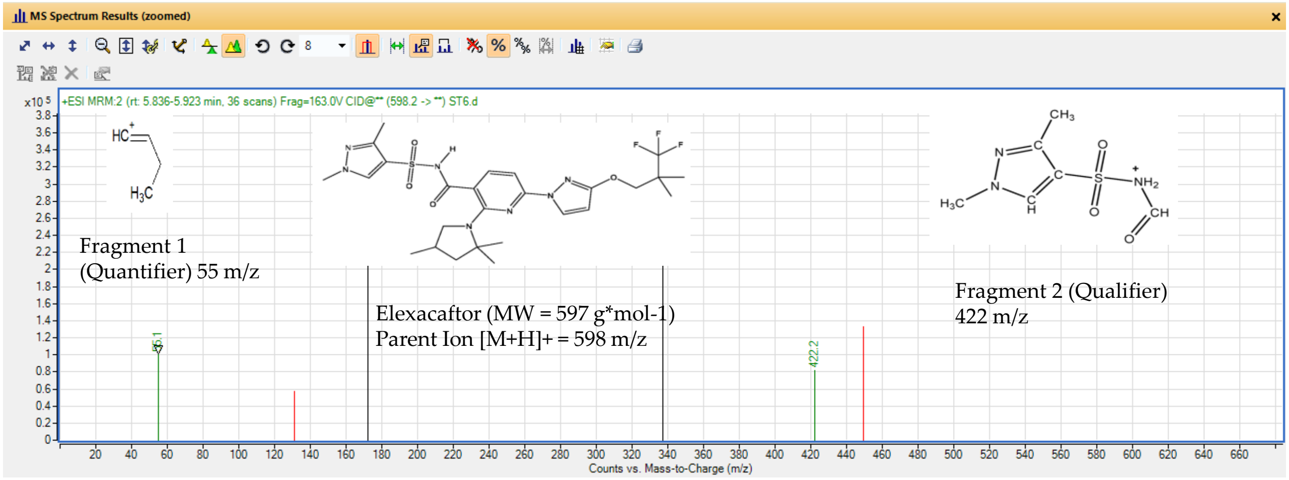

Supplementary Figure 2. Mass Spectrum results for Ivacaftor, Tezacaftor and Elexacaftor. Each panel reports the molecular structure of the parent ion and the fragments selected as quantifier and qualifier compounds.
