# Supplementary material for: Development and Validation of a New LC-MS/MS Method for Simultaneous Quantification of Ivacaftor, Tezacaftor and Elexacaftor Plasma Levels in Pediatric Cystic Fibrosis Patients
Source: Pharmaceuticals (Basel). 2025 Jul 10;18(7):1028. doi: 10.3390/ph18071028 (PMC12298270; doi:10.3390/ph18071028)
Supplement: Supplementary file 1 [file pharmaceuticals-18-01028-s001.zip › Supplementary Tables_R1.pdf]

Supplementary Table 1: System suitability values reported for Ivacaftor, Tezacaftor, Elexacaftor.

Compounds	Retention Time (min)	Resolution (Rs)	Capacity Factor (k′)	Peak Symmetry (As)	Tailing Factor (T)
**Tezacaftor**	4.603	11.45	4.3	0.81	1.1
**Ivacaftor**	5.343	7.31	5.2	1	1
**Elexacaftor**	5.876	7.31	5.8	0.89	1.1

Supplementary Table 2. Long-term – 80 °C stability for IVACAFTOR, TEZACAFTOR, ELEXACAFTOR

**IVACAFTOR**	**Time Point**	**Time 0**	**5 months**
	**Measured concentration for T0 (mean ± SD) n=3**	1.10 ± 0.39	0.96 ± 0.38
	**Stability (%) mean ± SD**		86.96 ± 3.98
	**Measured concentration for T4 (mean ± SD) n=3**	1.85 ± 0.22	1.53 ± 0.54
	**Stability (%) mean ± SD**		85.48 ± 19.83
**TEZACAFTOR**	**Time Point**	**Time 0**	**5 months**
	**Measured concentration for T0 (mean ± SD) n=3**	2.55 ± 1.50	2.32 ± 1.46
	**Stability (%) mean ± SD**		89.35 ± 4.87
	**Measured concentration for T4 (mean ± SD) n=3**	7.55 ± 1.34	6.15 ± 0.21
	**Stability (%) mean ± SD**		85.51 ± 11.87
**ELEXACAFTOR**	**Time Point**	**Time 0**	**5 months**
	**Measured concentration for T0 (mean ± SD) n=3**	10.38 ± 3.78	9.44 ± 3.68
	**Stability (%) mean ± SD**		90.55 ± 2.42
	**Measured concentration for T4 (mean ± SD) n=3**	15.40 ± 0.79	13.81 ± 3.49
	**Stability (%) mean ± SD**		89.18 ± 18.05

Supplementary Table 3 Comparison between bioanalytical method described here and previous published LC-MS/MS methods.

Bioanalytical method	Analyzed compounds	Instrumentation	Chromatographic column	Elution mode and Mobile phases used	Injection volume	Flow-rate (mL/min)	Run-time	Analysed matrix (required volume)	Calibration range (μg/mL); LLOQ (μg/mL)	Internal standard (IS) used	Validated Method (Yes or Not)
Method described here	Ivacaftor, Tezacaftor, Elexacaftor	UHPLC Agilent 1290 Infinity II apparatus (Agilent Technologies) coupled with 6470 Mass Spectrometry system (Agilent Technologies)	Luna Omega 1.60 μm Polar C18 (100 x 2.1 mm) Phenomenex	Gradient elution.<br><br>Mobile phase A (0.1 % formic acid in milli-q pure water);<br><br>Mobile phase B (0.1% formic acid in acetonitrile ACN and 25% of 2-propanol);	1.0 μL	0.4	10.50 mins	Plasma (50 μL)	0.1- 20.0; 0.05	Ivacaftor-d9, Tezacaftor-d4, Elexacaftor-d3	Yes

Viswanathan L. et al., 2022 [23]	Ivacaftor, Tezacaftor, Elexacaftor	API-4000 LC–MS/MS, Sciex	Supelco Ascentis Express RP C18, 2 cm × 2.1 mm, 2.7 μm	Gradient elution<br><br>Mobile Phase A (0.1% formic acid in water), Mobile Phase B (and 0.1% formic acid and 0.05% in MeOH/CAN 1:1)	n.r.	0.5	4.0 mins	Plasma	0.2- 0.01-10.0	d5-elexacaftor; d5-M23-elexacaftor	Yes
Bouazza N. et al., 2024 [25]	Ivacaftor, Lumacaftor	Waters Acquity UPLC coupled with Xevo TQD triple-quadrupole MS	Waters BEH C18 (50 x2.1, 1.7 μm)	Gradient elution, Mobile Phase A (0.05 % FA in water), Mobile Phase B (0.05 % FA in ACN)	10 μL	n.r.	7 min	Plasma	0.075-20 mg/L 0.3-	D18-ivacaftor	Yes
Yuan et al., 2021 [32]	Ivacaftor	Agilent 1200 HPLC system and Agilent 6460 QQQ Mass Spectrometer	A Kinetex C18 (2.6 mm, 100 Å, 50 mm, 3 mm) column (Phenomenex)	Gradient elution.<br><br>Mobile phase A (0.1 % formic acid in milli-q pure water); Mobile phase B (0.1% formic acid in acetonitrile (ACN)	10.0 μL	0.4	6 mins	Rat Plasma or Cells Culture (1200 μL)	0.1-10 μM; 1.25 μM	None	Yes

Reyes-Ortega et al., 2020 [33]	I Ivacaftor, Tezacaftor, Elexacaftor, Ivacaftor−carboxylate/ ivacaftor-M6 metabolite, hydroxymethyl−ivacaftor/ivacaftor-M1	Triple-quadrupole Shimadzu 8030 LC-MS	C8 column (Phenomenex; 2.6 µm, 100 Å; 50 × 2.1 mm) with the guard column C8 (Phenomenex; 2.1 mm ID Columns, 3/Pk)	Gradient elution.<br><br>Mobile phase A (0.1 % formic acid in water); Mobile phase B (0.1% formic acid in acetonitrile (ACN)	2.0 µL	0.5	5.5 mins	Plasma	0.0001-1.0	Ivacaftor−carboxylate/ ivacaftor-M6 metabolite, hydroxymethyl−ivacaftor/ivacaftor-M1	Yes
Vonk SEM et al. 2021 [34]	Ivacaftor, hydroxymethyl ivacaftor, Ivacaftor carboxylic acid, Lumacaftor, Tezacaftor	LC-30 Nexera (Shimadzu) coupled with 5500 QTrap mass spectrometer (SCIEX)	A HyPURITY C18 HPLC (50 · 2.1 mm, 1.9 mm) column (Thermo Scientific, Waltham, MA)	Gradient elution<br><br>Mobile Phase A (0.1% vol/vol formic acid and 0.05% vol/vol ammonium formate in ultrapure water), Mobile Phase B (and 0.1% vol/vol formic acid and 0.05% vol/vol ammonium formate in ACN)	2 µL	0.5	6.0 mins	Plasma and Sputum (20 µL)	0.01-10.0	Ivacaftor-d9	Yes

Pigliasco R. et al., 2023 [35]	Ivacaftor, Tezacaftor, Elexacaftor	Thermofisher Quantiva triple-quadrupole MS coupled to an Ultimate 3000 UHPLC	Accucore Polar Premium (50 mm x 2.2 mm, 2.6 μm) Thermofisher	Gradient elution.<br><br>Mobile phase A (0.1 % formic acid in milli-q pure water); Mobile phase B (0.1% formic acid in acetonitrile ACN)	5.0 μL	0.5	5 min	Plasma (50 μL)	0.008-12 μM	Ivacaftor-d9, Tezacaftor-d4, Elexacaftor-d3	Yes
Schneider EK et al., 2017 [36]	Ivacaftor, M1, M6 and Lumacaftor	Shimadzu 8030 LC–MS system coupled with the 8030 triple quadrupole mass spectrometer	C8; 2.6 μm; 100 Å; 50 x 2.1 mm, Guard column HPLC In-Line Filter 0.5 μm; Depth Filter x 0.004 inID	Gradient elution, Mobile Phase A (0.1 % FA in water), Mobile Phase B (100 % ACN)	5 μL	0.5 mL/min	6 mins	Plasma	0.01-10.0	IVA, M1, M6 and LUMA	Yes
Habler K et al, 2021 [37]	Ivacaftor, Lumacaftor, Tezacaftor, Elexacaftor, IVA-M1, IVA-M6, TEZ-M1	Waters Acquity UPLC coupled with Xevo TQ-XS Tandem Mass Spectrometer	Waters Oasis HLB column (25 μm, 2.1x20 mm, SPE Security Guard Column C18 (4 x 3.0 mm, Phenomenex)	Gradient elution, Mobile Phase A1 (Methanol/water 10/90), Mobile Phase A2 Methanol/water 90/10), Mobile Phase B1 (methanol)	n.r.	0.4 mL/min	5 min	Serum	0.15-7.5 for IVA; 0.30-15.0 for IVA-M1, TEZ and ELX; 0.08–3.75 for IVA-M6; 0.80-40.0 for LUM; 0.50-25.0 for TEZ-M1;	TEZ-d4, ELX-d3, LUM-d4, IVA-d4	Yes

Zheng Y et al, 2024 [38]	Ivacaftor, Lumacaftor, Elexacaftor, Tezacaftor, IVA-M1, IVA-M6	Waters Acquity UPLC coupled with Xevo TQD triple-quadrupole mass-spectrometer	Waters BEH (ethylene bridge hybrid) C18 analytical column (50 ×2.1 mm, 1.7 μm)	Gradient elution, Mobile Phase A (0.05 % FA in water), Mobile Phase B (0.05 % FA in ACN)	10 μL	0.5 mL/min	7 min	Plasma	0.075-20.000 mg/L for LUM, TEZ and ELX, from 0.053-14.000 mg/ L for IVA, and from 0.024-6.500 mg/L for IVA-M1 and IVA-M6	IVA-d18, LUM-d4	Yes
Vonk SEM et al, 2024 [39]	Elexacaftor, Elexacaftor-M23, Tezacaftor, Tezacaftor-M1, Ivacaftor, Ivacaftor Carboxylate (IVA-M6), and Hydroxymethyl Ivacaftor (IVA-M1)	LC-30 Nexera (Shimadzu) coupled with 5500 QTrap mass spectrometer (SCIEX)	HyPURITY C18 HPLC (50 · 2.1 mm, 3.0 mm) column (Thermo Scientific)	Gradient elution Mobile Phase A (0.1% vol/vol formic acid and 0.05% vol/vol ammonium formate in ultrapure water), Mobile Phase B (and 0.1% vol/vol formic acid and 0.05% vol/vol ammonium formate in ACN)	2 μL	0.5 mL/min	6.5 min	DBS samples	0.01-10 mg/L for Elexacaftor, Elexacaftor-M23, Tezacaftor Ivacaftor, Ivacaftor Carboxylate (IVA-M6), and Hydroxymethyl Ivacaftor (IVA-M1), 0.025-10 mg/L for Tezacaftor-M1	Ivacaftor-D9, Elexacaftor-D3, and Tezacaftor-D4	Yes

A. Mireille A. Wessels et al, 2025 [40]	ivacaftor, tezacaftor, elexacaftor, lumacaftor, hydroxymethyl ivacaftor (IVA-M1), tezacaftor M1, and N-desmethylelexacaftor (ELEXA-M23)	Thermo Fisher Scientific Quantiva® tandem quadrupole mass spectrometer coupled with Thermo Fisher Scientific Vanquish® UHPLC-system (Thermo Fisher Scientific, San Jose, CA, USA)	Thermo Scientific Accucore C18 column (2.6 µm, 50 ×2.1 mm)	Mobile phase A (ammonium formate 20 mM (adjusted to pH 3.5 with formic acid) and mobile Phase B (100 % acetonitrile)	5 µL	1.0 mL/min	2.1 min	Plasma (10 µL)	0.010 to 10 mg/L for ivacaftor, IVA-M1 and ELEXA-M23; 0.025 to 25 mg/L for elexacaftor and tezacaftor; 0.050 to 50 mg/L for tezacaftor M1; 0.100 to 100 mg/L for lumacaftor	$^{2}H_{9}$-ivacaftor; $^{2}H_{4}$-tezacaftor; $^{2}H_{3}$-elexacaftor.	Yes

For reference numbers see the main manuscript.
